# Evaluating the Translucency, Surface Roughness, and Cytotoxicity of a PMMA Acrylic Denture Base Reinforced with Bioactive Glasses

**DOI:** 10.3390/jfb15010016

**Published:** 2023-12-31

**Authors:** Abdulaziz Alhotan, Zbigniew Raszewski, Katarzyna Chojnacka, Marcin Mikulewicz, Julita Kulbacka, Razan Alaqeely, Amani Mirdad, Julfikar Haider

**Affiliations:** 1 Department of Dental Health, College of Applied Medical Sciences, King Saud University, P.O. Box 10219, Riyadh 12372, Saudi Arabia; 2 SpofaDental, Markova 238, 506-01 Jicin, Czech Republic; 3 Department of Advanced Material Technologies, Faculty of Chemistry, Wroclaw University of Science and Technology, Smoluchowskiego 25, 50-372 Wroclaw, Poland; 4 Department of Dentofacial Orthopaedics and Orthodontics, Division of Facial Abnormalities, Wroclaw Medical University, Krakowska 26, 50-425 Wroclaw, Poland; 5 Department of Molecular and Cellular Biology, Faculty of Pharmacy, Wroclaw Medical University, Borowska 211A, 50-556 Wroclaw, Poland; 6 Department of Immunology, State Research Institute Centre for Innovative Medicine, Santariškių 5, 08410 Vilnius, Lithuania; 7 Department of Periodontics, College of Dentistry, King Saud University, P.O. Box 10219, Riyadh 12372, Saudi Arabia; 8 Department of Engineering, Manchester Metropolitan University, Manchester M1 5GD, UK

**Keywords:** acrylic resin, bioactive glass, cytotoxicity, elemental analysis, ions release, surface roughness

## Abstract

The colonisation of the surface of removable acrylic dentures by various types of microorganisms can lead to the development of various diseases. Therefore, the creation of a bioactive material is highly desirable. This study aimed to develop a denture base material designed to release bioactive ions into the oral environment during use. Four types of bioactive glasses (BAG)—S53P4, Biomin F, 45S5, and Biomin C—were incorporated into the PMMA acrylic resin, with each type constituting 20 wt.% (10 wt.% non-silanised and 10% silanised) of the mixture, while PMMA acrylic resin served as the control group. The specimens were subsequently immersed in distilled water, and pH measurements of the aqueous solutions were taken every seven days for a total of 38 days. Additionally, surface roughness and translucency measurements were recorded both after preparation and following seven days of immersion in distilled water. The cytotoxicity of these materials on human fibroblast cells was evaluated after 24 and 48 h using Direct Contact and MTT assays. Ultimately, the elemental composition of the specimens was determined through energy-dispersive X-ray (EDX) spectroscopy. In general, the pH levels of water solutions containing BAG-containing acrylics gradually increased over the storage period, reaching peak values after 10 days. Notably, S53P4 glass exhibited the most significant increase, with pH levels rising from 5.5 to 7.54. Surface roughness exhibited minimal changes upon immersion in distilled water, while a slight decrease in material translucency was observed, except for Biomin C. However, significant differences in surface roughness and translucency were observed among some of the BAG-embedded specimens under both dry and wet conditions. The composition of elements declared by the glass manufacturer was confirmed by EDX analysis. Importantly, cytotoxicity analysis revealed that specimens containing BAGs, when released into the environment, did not adversely affect the growth of human gingival fibroblast cells after 48 h of exposure. This suggests that PMMA acrylics fabricated with BAGs have the potential to release ions into the environment and can be considered biocompatible materials. Further clinical trials are warranted to explore the practical applications of these materials as denture base materials.

## 1. Introduction

The construction of the base for removable dentures currently relies primarily on the use of polymethyl methacrylate (PMMA). This choice is attributed to its ease of processing and cost-effectiveness, consisting of powder and liquid forms [1]. When combined, these components create a pliable dough that can be readily moulded using a single-use gypsum mould following wax boiling. Subsequently, the material undergoes heat curing and polishing [1]. PMMA offers several advantages, including biological inertness, high translucency, and compatibility with the oral cavity environment (provided proper polymerisation is achieved and residual monomers are not released) [2].

Over time, various types of microorganisms can colonise removable acrylic dentures, adversely affecting the health of users and even leading to caries in any remaining teeth [2]. Consequently, there is a pressing need to develop a material capable of releasing ions that can alter the pH of the oral environment, thereby preventing tooth decay [3]. The development of such a material is complex and requires extensive research. Often, the surface of prostheses is colonised by C. albicans, and currently employed oral antifungal drugs like amphotericin B, nystatin, and miconazole, as well as systemic antifungal medications, have limited efficacy [4].

The literature presents various methods for creating a material suitable for dental prostheses that can deter microorganism colonisation. One approach involves incorporating substances with bacteriostatic properties, such as silver nanoparticles [5,6], titanium dioxide nanoparticles [7,8], graphene and silver nanoparticles [9], silanised zinc oxide nanoparticles [10], zirconium dioxide nanoparticles [11,12], SiO_2_ nanoparticles [13], or methacrylate components with active chains like 2-methacryloyloxyethyl phosphorylcholine, Dimethylaminohexadecyl methacrylate [14], zinc dimethacrylate [15], zirconium methacrylate (ZM), tin methacrylate (TM), and di-n-butyldimethacrylate-tin [16,17]. However, there is no commercial product on the market with the desired properties.

Bioactive glasses (BAGs) undergo gradual hydrolysis under the influence of water or bodily fluids, releasing various types of ions (e.g., calcium or fluorine). Calcium cations can increase pH, thus inhibiting the growth of microorganisms. Fluorine anions have proven bactericidal properties [4]. BAGs have been tested in various dental materials, including composite materials, and even in commercial products such as toothpaste. Composite materials contain cross-linked resins, so the method of release of ions from bioactive glasses may differ from that in the case of acrylic materials [4]. These glasses can release calcium ions with alkalising properties from their surface [4]. One innovative concept involved using glass ionomer cement combined with acrylic acid to form a gel, which was then added to the acrylic material at concentrations ranging from 5 wt.% to 20 wt.% [4].

The novelty of this series of works lies in the use of this type of material in acrylic materials based on polymethyl methacrylate. Because this material is chemically different from the composite materials used in dentistry, it presents new avenues for research. Previous studies have documented tests on acrylic specimens modified with different types of bioactive glasses, including Biomin C, Biomin F, S53P4, and 45S5 [18]. These tests evaluated mechanical properties like flexural strength, sorption, and solubility. Additionally, the study examined the release of phosphorus, calcium, fluoride, and silicon ions over a 60-day period [18]. However, further comprehensive research into its functional properties and safety is necessary for the material to be deemed suitable for medical use. Thus, this study constitutes a continuation, assessing additional parameters of bioactive materials, such as surface roughness, translucence, cytotoxicity, EDX analysis and changes in pH when immersed in distilled water.

The initial hypothesis for this investigation posited that PMMA resin material, when augmented with bioactive glasses, remains acceptable in terms of transparency, surface roughness, cytotoxicity, and its capability to increase water pH through the release of alkaline ions. Since there may be various types of reactions between the polymer and glass particles, it is necessary to additionally test the resulting system [19].

## 2. Materials and Methods

### 2.1. Materials Preparation

Bioactive glass specimens with the compositions outlined in Table 1 were sourced from Cera Dynamic (Kent, England). To obtain better mechanical properties and slow down the speed of ion release, the acrylic resin (Superacryl Plus, SpofaDental, Jicin, Czech Republic, pink colour, batch number 1045678) was mixed with 20 wt.% BAGs. Each specimen contains a mixture of 10% silanised glass and 10% non-silanised glass.

Some of the material had undergone silanisation, with a detailed procedure described in previous research [20]. Subsequently, Superacryl Plus acrylic powder (SpofaDental, Jicin, Czech Republic) was mixed with 10% silane-modified bioactive glass and 10% surface-unaffected glass. This mixture was processed in a ball mill (Jezirska Porcelanka, Czech Republic) for 30 min to achieve homogenisation. For each glass with acrylic resin, a batch of 200 g of powder was prepared (800 g total) at a time. For the most part, the powder was combined with Superacryl Plus monomer (SpofaDental, Jicin, Czech Republic) and allowed to maturate covered in a glass vessel for 10 min. This duration is referred to as the “dough time” during which the material no longer adheres to the hands. At this stage, the material was placed in metallic cylinder-shaped moulds of various diameters and thicknesses, depending on the specific tests to be conducted. The material was thermally polymerised in the moulds for 60 min at 100 °C. Upon completion of the curing process, the material was removed from the moulds. All specimen surfaces were smoothed using carbide burs number 13,052 for acrylic (DFS, Cologne, Germany) and a micromotor (Kavo K7) with a rotation speed of 3000 rpm for 5 min. They were subsequently further smoothed using 100-grit sandpaper (Saint Gobain, Kolo, Poland) before undergoing testing.

In addition to specimens of acrylic modified with bioactive glasses, reference specimens of pure Superacryl Plus (PMMA, SpofaDental, Jicin, Czech Republic) were also prepared. For the pH change test, 5 specimens of each material were cured (25 specimens in total). For the surface smoothness test, 6 specimens were prepared (36 specimens in total). In the case of transparency tests, 6 specimens were produced for each research group (30 specimens in total for each group), 6 specimens were designated for cytotoxicity measurements (30 specimens in total), and 3 specimens were selected for EDX analysis tests (15 specimens in total).

### 2.2. pH Analysis

The ability of bioactive materials to regulate pH levels in the mouth’s environment is a critical aspect of their functionality [5,6]. One way to prevent enamel demineralisation and inhibit the growth of microorganisms is by raising the pH of the environment to above 5–5.5 [20]. To assess whether the combination of acrylic specimens and bioactive glass can influence pH, PMMA (the reference material) and acrylics modified with 20% BAGs were placed in containers, each containing 10 mL of distilled water. For the test, five discs, each measuring 6 mm in diameter and 2 mm in thickness, were used for each material, resulting in a total of 25 specimens. These containers with acrylic discs were then placed in a laboratory drying oven set at 37 °C. At specific time intervals (initially after 3 days and then every 7 days thereafter), the specimens were removed from the drying oven. After cooling to room temperature, their pH levels were measured using a Mettler Toledo pH 7110 SET instrument (Mettler Toledo sro, Prague, Czech Republic) equipped with a SenTix^®^ 81 electrode and 3 mol/L KCl buffer solutions. The instrument required daily calibration, which was performed using pH = 7 buffers from the same company. Following each pH measurement, the entire solution was removed from the container, and the container was rinsed with distilled water (DW). The specimen was then dried using filter paper, and a new portion of DW (pH = 5.6) was added.

The first measurement point was determined after 3 days of specimen immersion in distilled water, which is why previous tests [20] observed a change in the colour of the specimen. This change could indicate the release of ions on the surface of the acrylic material. Subsequent measurements were taken every 7 days.

### 2.3. Surface Roughness

The smoothness of the prosthesis’s surface plays a crucial role in preventing the adhesion of microorganisms, a topic extensively covered in the literature. Consequently, assessing the roughness of a new material incorporating BAGs is imperative. A detailed description of this test was presented by Moslehifard, et al. [21]. Specimens measuring 10 mm in diameter and 2 mm in thickness were polymerised using the method outlined above. Six specimens from each material (30 in total) were selected for testing.

To measure the average surface roughness (Ra) of each of the six specimens from every material, a noncontact surface profilometer (Contour GT-X, ver.5.30, Bruker, Billerica, MA, USA) was employed. Measurements were conducted both under dry conditions and after 7 days of storage in distilled water. Each specimen was securely affixed to the automated x-y stage for scanning. The scanning process parameters included a ×5 nano lens, a ×1 field of view, ×1 scan speed, and ×0.1 stage speed, utilising Vision Map 64 software. Readings were obtained at three distinct locations on each specimen’s surface, and the average roughness (Ra) for each specimen was determined.

### 2.4. Translucency

Materials used for dental prostheses must possess a certain level of transparency to resemble the gingival mucosa [1]. To evaluate this transparency, translucency measurements were conducted using a method extensively described by.

For each material tested, six specimens underwent reflectance measurements under both dry conditions and after immersion in distilled water for 7 days. This was achieved using an automated variable specimen illumination system and a spectrophotometer (LabScan XE, Hunter Associates Laboratory Inc., Reston, VA, USA). Prior to conducting the measurements, the spectrophotometer was calibrated following the manufacturer’s instructions, which involved using a black trap and a white tile. The translucency parameters (*TP*) were subsequently calculated by determining the differences in the L*, a*, and b* values recorded against white (*W*) and black (*B*) backgrounds, employing the formula provided below [22]:

$$TP=\sqrt{{\left(L_{B}^{*}-L_{W}^{*}\right)}^{2}+{\left(a_{B}^{*}-a_{W}^{*}\right)}^{2}+{\left(b_{B}^{*}-b_{W}^{*}\right)}^{2}}$$

where L* represents lightness, a* corresponds to the range of colours from red to green, and b* pertains to the range from yellow to blue.

### 2.5. Cytotoxicity

Subsequently, the impact of the materials prepared in Section 2.1 on cell cultures was investigated [23]. To assess the cytotoxicity, human gingival fibroblasts (HGF) were utilised. This primary cell culture originated from a fragment of gingival tissue, as previously detailed by Nowakowska, et al. [23], and was cultivated under controlled laboratory conditions. The cells were cultured in DMEM (Dulbecco’s Modified Eagle’s Medium, Sigma-Aldrich, Poznan, Poland) supplemented with 10% FBS (Sigma-Aldrich, Poznan, Poland), Penicillin/Streptomycin (Sigma, Poznan, Poland), and 1% GlutaMAXTM-I (Gibco, New York, NY, USA) in 25 cm^2^ flasks (Zeit Buddels, Sarstedt, Germany). To facilitate their development, the cells were incubated in a humid environment at 37 °C with 5% CO_2_.

#### 2.5.1. Assessment of Cell Morphology and Migration as Cytotoxicity Indicators

This method allows for the direct observation of how the tested specimens with bioactive glasses affect target cells. The cells were cultured in a standard Dulbecco’s Modified Eagle Medium (DMEM, Sigma, Poznan, Poland), along with the experimental products, including four bioactive glasses and PMMA as a reference specimen. For microscopic examination, the test articles were placed in a 24-well plate (Nunc™ Multrays with UpCell™: 174901, Fisher Scientific, Warsaw, Poland), followed by seeding HGF cells in a culture medium at a density of 104 cells per well. This setup was maintained in a humid environment at 37 °C with 5% CO_2_. Observations under the microscope were conducted after 1 h and 24 h of exposure to the tested products using an optical microscope (Leica DMi 1, produced by CellService, Poznan, Poland). A more detailed description of these tests can be found in Nowakowska, et al. [23].

#### 2.5.2. Cytotoxicity Test—Direct Contact and MTT Assay

Four specimens, each with a minimum diameter of 5 mm and a thickness of 1 mm, were produced for cytotoxicity tests. The impact of the modified acrylic specimens on gingival fibroblasts was assessed at 24 and 48 h of direct contact using the PrestoBlue^®^ test. Before exposure, the test acrylic discs were placed in a 24-well plate (Sarstedt, Germany), and HGF cells were seeded in a culture medium at a density of 1104 cells per well. After 24, and 48 h, the culture medium was collected for evaluation tests conducted by PrestoBlue^®^ measurements.

PrestoBlue^®^ is a pre-prepared, cell-permeable resazurin-based solution that serves as an indicator of cell viability, utilising the reducing capacity of living cells to quantify cell proliferation. The absorbance of this dye was measured using a multi-plate reader (GloMax^®^ Discover, Promega, Madison, WI, USA) at a wavelength of 560 nm to detect the proper colour intensity. Each test specimen underwent exposure in three replicates. The results derived from these measurements, representing cell viability, were calculated in comparison to the values from a similar test involving control cells that had no contact with the test materials. A more detailed description of this test can be found in the publication by Saczko, et al. [24], and average values were used in the calculations [11].

### 2.6. Analysis of the Composition of the Elements Inside the Specimen

The specimens were chosen at random and placed into the Scanning Electron Microscope (SEM)/EDX (Bruker, Billerica, MA, USA) to be imaged by a secondary electron detector with an acceleration voltage of 20.0 kV. The specimens were mounted on aluminium stubs before being sputter-coated with a wafer-thin gold layer [25]. Elemental analysis was performed using Energy-dispersive X-ray spectroscopy (EDX), which allows for the determination of the concentration of specific elements in the specimens because each element has a unique set of peaks on its emission spectrum. The existence of all elements was compared with the information provided by the Cera Dynamic supplier of the glasses.

### 2.7. Statistical Analysis

The statistical analysis was carried out using SPSS Inc. (Version 27, Chicago, IL, USA). The Shapiro-Wilk test was used to determine the normality of each variable, and the average and standard deviation were computed. As the variables displayed a normal distribution, a parametric analysis was conducted. To analyse the variations in surface roughness, pH, and transparency (independent variables) among the materials concerning storage time, a two-way ANOVA was utilised, followed by Tukey’s post hoc test (*p* ≤ 0.05). Additionally, to evaluate the differences at each storage time (comparison between dry and the 7-day period) for each material, an independent t-test was applied (*p* ≤ 0.05).

## 3. Results

**Figure 1:**
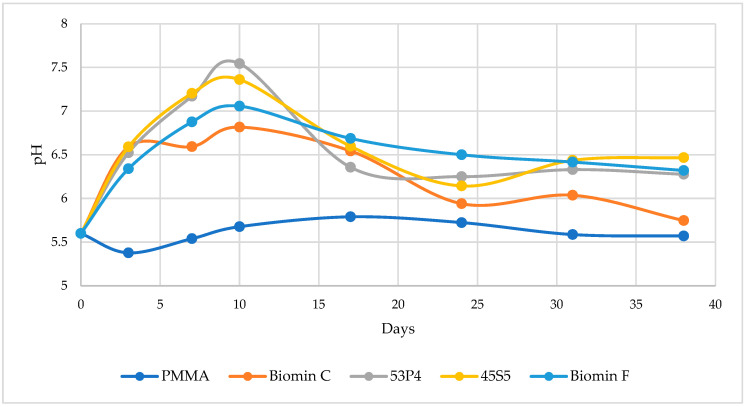
Change in pH after storage in distillate water at 37 °C for 38 days.

Figure 1 illustrates the time-dependent pH changes for each acrylic specimen incorporated with BAGs and stored in distilled water. In all tested specimens, the maximum pH increase occurred after 10 days of water immersion. Subsequently, the release of alkali ions gradually decreases but remains observable. Distilled water alone serves as the reference point with a pH of 5.6.

**Figure 2:**
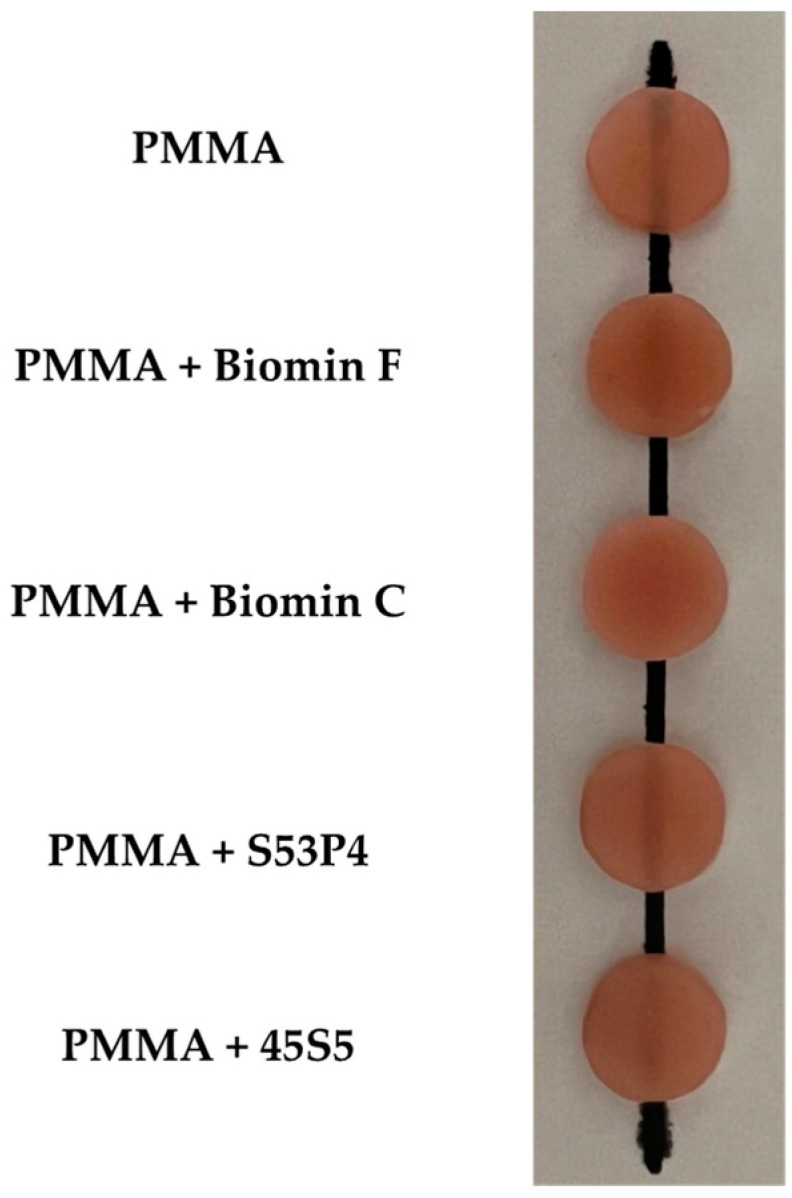
The specimens from each group appearance.

Table 2 presents the results concerning changes in surface roughness and surface translucency for specimens immersed in water for 7 days. The test results indicate that the material becomes less translucent (Figure 2) when augmented with bioactive glasses compared to PMMA. This reduction in translucency is particularly noticeable when using Biomin C glass, resulting in a translucency value of 4.18 ± 0.4.

**Figure 3:**
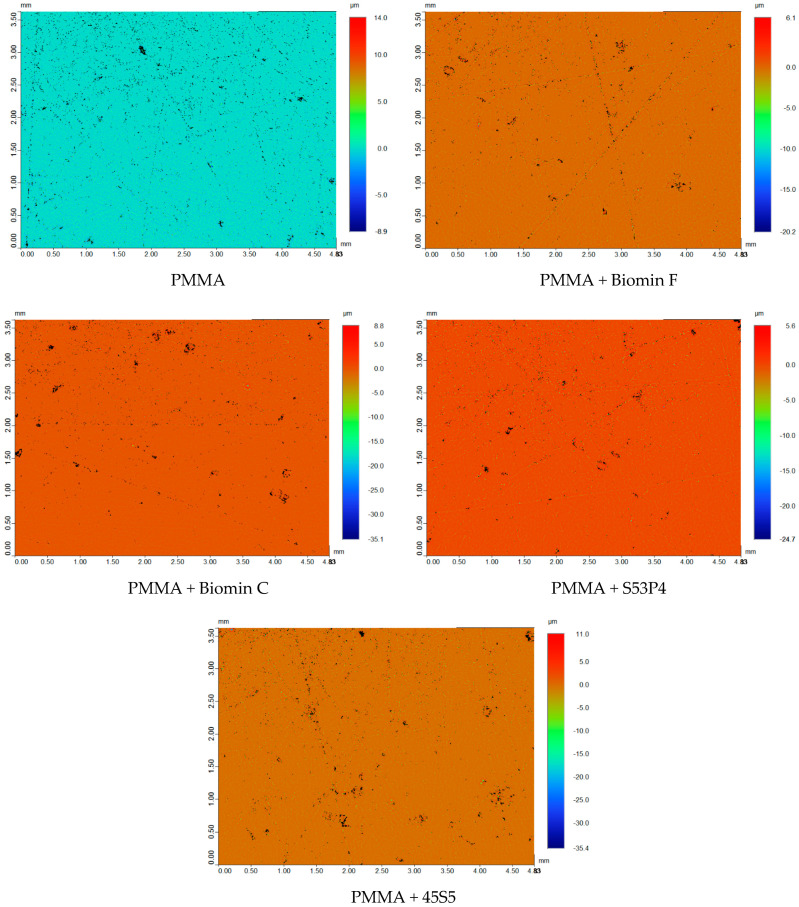
Profilometer images of the tested materials.

Surface roughness is observed in all specimens, whether in dry or wet conditions. The specimens with the lowest roughness are found in the PMMA resin and when Biomin F and Biomin C are added to PMMA; however, this difference is not statistically significant. Glass 45S5 and S53P4 contribute to greater surface roughness than the pure polymethyl methacrylate specimen. After 7 days of storage in water, there is a slight increase in surface roughness, although this difference is not statistically significant when compared to the specimen with pure PMMA. For specimens that include bioactive glasses, darker discolouration can be observed in Figure 3, indicating clusters of undispersed fillers.

**Figure 4:**
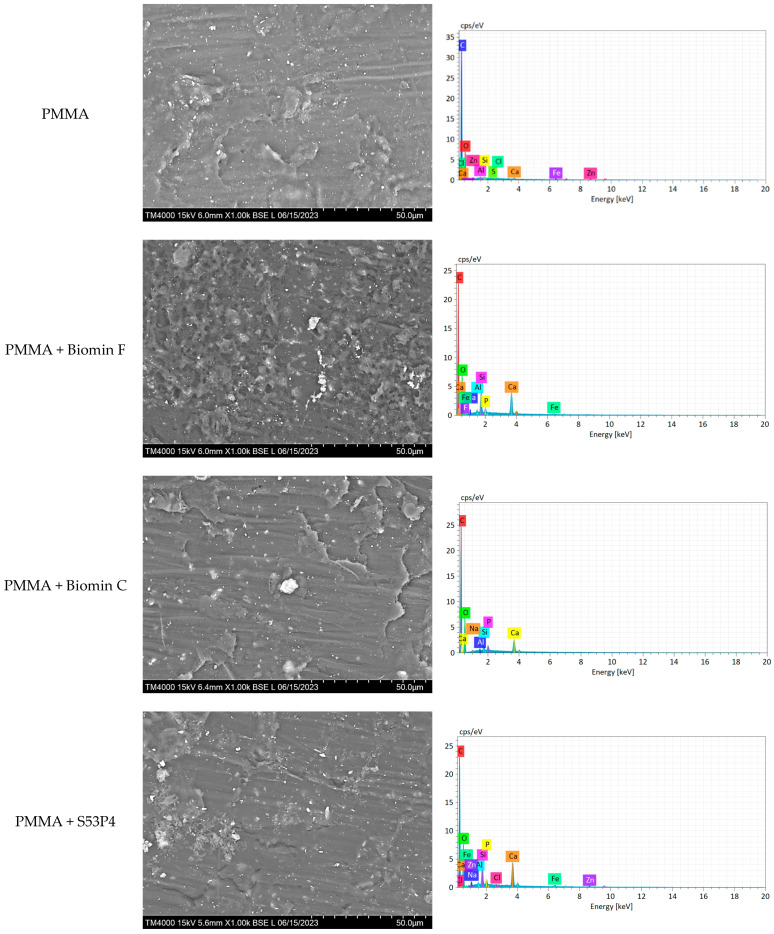

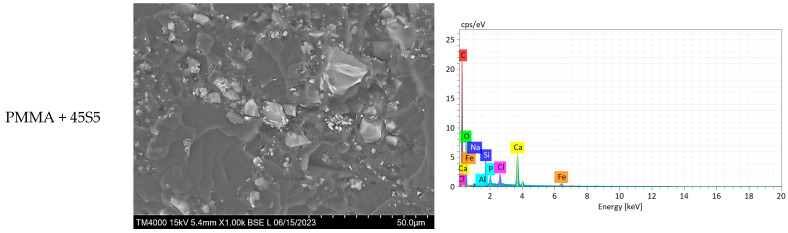
Images of the surface and elemental spectra of the specimens.

The surface SEM images and elemental composition of the prepared specimens are presented in Figure 4 and Table 3. Surface images of PMMA with the addition of bioactive glasses reveal the presence of lighter crystals of bioactive glasses with a size of 1 μm distributed within the material’s structure. However, in some regions, clusters of unmixed BAGs of approximately 20 μm are visible (e.g., 45S5 and Biomin C).

**Figure 5:**
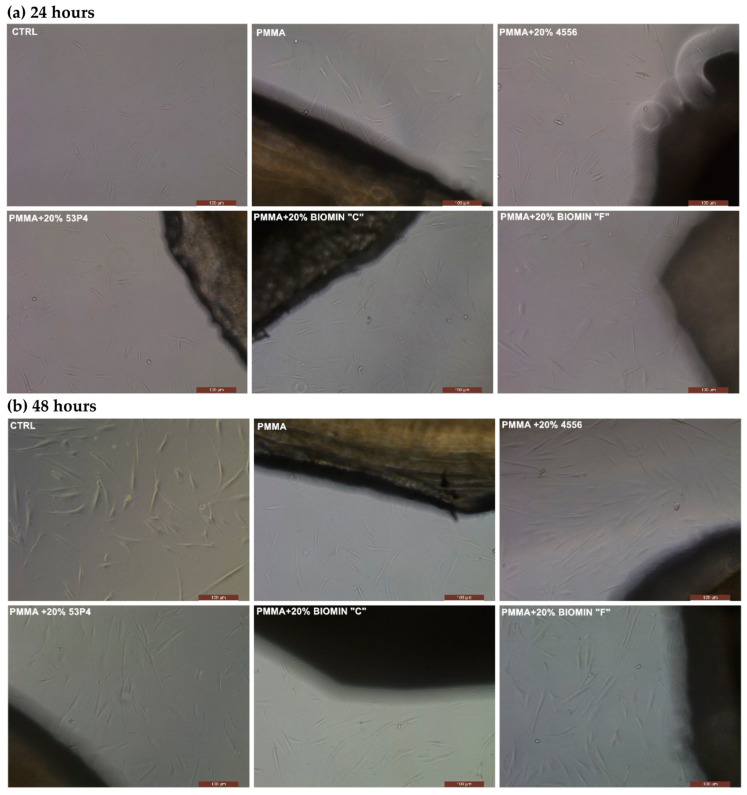
Human cells in direct contact with acrylic specimens containing 20% bioactive glasses. CTRL specimens with direct contact with the culture medium.

**Figure 6:**
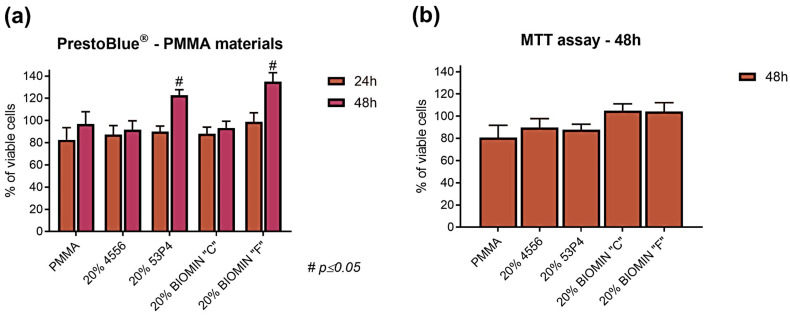
Viability of human cannon fibroblast cells in direct contact with acrylic specimens containing bioactive glasses measured by (**a**) PrestoBlue® assay after 24 and 48 h, and (**b**) MTT assay after 48 h. Statistically significant for # *p* < 0.05 in comparison to untreated CTRL, and 24 vs. 48 h.

Figure 5 depicts the appearance of human gingival fibroblast cells in direct contact with acrylic material specimens containing 20 wt.% bioactive glass. After 24 and 48 h of contact, the size, shape, and number of cells remain unchanged. Figure 6 presents the percentage of cell viability using the Presto Blue test, which stains live cells. In the case of Biomin F and S53P4, cell viability exceeds 80%, and after 48 h, it exceeds 120% of the number of reference cells. Other glasses and PMMA also do not show significant changes in cell viability. Similar results were also obtained in the second MTT test.

## 4. Discussion

The hypothesis formulated for this study received only partial support. Upon the incorporation of bioactive glasses, acrylic materials demonstrated the capacity to release alkaline ions, resulting in an elevation of the pH level in distilled water without exhibiting cytotoxicity. However, the anticipated outcome concerning surface roughness was not corroborated; instead, it escalated, accompanied by a decrease in translucency. This indicates that materials augmented with bioactive glasses possess lower translucency compared to pure PMMA.

Elemental analysis conducted revealed a predominant carbon concentration (over 50%) originating from PMMA, with zinc serving as the white pigment in the composition. The minimal presence of chlorine (Cl), calcium (Ca), and sulphur (S) corresponds to the components of the organic red pigment. The inclusion of iron in the composition serves the purpose of yellow and brown pigments, simulating gingival colour. A comparison between Table 1 and Table 3 reveals that all elements provided by the manufacturer for the glasses were identified using EDX technology (e.g., Cl for Biomin C, F for Biomin F, and Na, P, Si for other glasses). Traces of aluminium (Al) are attributed to the metal forms employed during specimen preparation, which are constructed from aluminium.

In specimens containing S53P4 bioactive glass, an increase in surface roughness was observed after immersion in water, shifting from 0.364 ± 0.04 µm to 0.372 ± 0.06 µm. This implies that ions can migrate through the resin structure and accumulate on the surface. Greater surface roughness translates into a larger surface area. This, in turn, allows for greater water adsorption, which facilitates the gradual hydrolysis of glasses and the migration of ions to the specimen surface. However, this phenomenon was not universally observed for all types of glasses, suggesting that different glasses may undergo hydrolysis at varying rates, resulting in ions migrating to the acrylic surface over different timeframes. The surface of dental dentures can be made as smooth as possible to prevent microorganism colonisation. Although the addition of bioactive glasses causes a slight increase in surface roughness, which may facilitate biofilm growth, the presence of calcium ions on the surface could help inhibit this process. This observation aligns with the findings reported by Tsutsumi, et al. [4]. According to [4], who examined the bioactivity of pre-reacted glass in acrylic resins, it was feasible to reduce C. albicans adhesion to removable acrylic denture bases that incorporated 5–35 wt.% filler. This property is highly desirable from a clinical perspective. Bioactive glasses, such as 45S5, have demonstrated some antimicrobial efficacy by releasing sodium ions (Na^+^) and calcium ions (Ca^2+^) when suspended in an aqueous solution, thereby creating an environment with a high pH level that is inhospitable to certain microorganisms. Furthermore, experimental evidence suggests that silica released from these glasses contributes to their antibacterial effect by directly inhibiting bacterial viability and acting as surfactants at solid–liquid interfaces [26].

During the preparation of dentures, thorough polishing is essential. However, over time and through the use of dentures, the surface of acrylic resins may change due to disinfection processes [27,28] and prolonged exposure to the oral environment. Therefore, it is crucial to maintain proper denture base polishing. Dental professionals can perform this task during denture maintenance procedures, such as rebasing or the addition of teeth [27,29].

Gad, et al. [26] also delved into the impact of bioactive glass BAG on C. albicans adhesion to acrylic denture base material. Their observations revealed significantly higher surface roughness and surface hardness in the tested specimens with bioactive glass. The addition of bioactive glass can increase surface hardness because it functions as an inorganic filler, a phenomenon we previously noted in our prior publication [18]. In this study, the leaching of alkali ions from acrylic resins, leading to an increase in pH, was also observed. The maximum elution occurred after 10 days, during which specimens with S53P4 bioactive glass raised the water pH from 5.5 to 7.54. This observation aligns with our previous findings [18], where all PMMA resins with 20 wt.% bioactive glasses demonstrated ion release over 60 days. The mechanism behind particle leaching involves the extraction of residual monomers by water, resulting in voids on the surface and within the polymer matrix. These voids facilitate the diffusion of recharging solutions deeper into the polymer matrix, enhancing ion release and storage potential [30]. Furthermore, the absorption of solutions by hydrophilic PMMA denture base materials, as described in the literature, can increase ion uptake and release from dissolved glass fillers [26].

In the context of denture base materials, the aesthetic appearance of the final device is of paramount importance to patient perception. Ideally, the gingival portion of the denture should be transparent to achieve a natural tissue-like effect [1]. Therefore, it is valuable to evaluate the translucency of acrylic polymers after modification with materials that can enhance biological or mechanical properties. Sagadevan, et al. [31] investigated these properties for acrylic resin containing zirconium nanoparticles and nano cellulose fibres, noting a reduction in translucency for specimens with 5% nanomaterial concentration, although it remained within acceptable limits. Similar results were obtained in this study, with certain BAG glasses having a more pronounced impact on translucency after incorporation into PMMA and immersion in distilled water, especially Biomin C. Discs with a 20 wt.% concentration of this filler exhibited significantly reduced transparency compared to the reference specimens made from PMMA, resulting in a much whiter appearance. Additionally, the specimens containing S53P4 BAG displayed lower transparency compared to PMMA material. This can be attributed to the compatibility of refractive indices between PMMA (1.46) and the glasses [32]. It has been demonstrated that with proper distribution and homogeneous mixtures, improved optical properties can be achieved by incorporating various fillers into PMMA resins. The small nanoparticle size allows them to fill gaps between polymer chains, creating a homogeneous PMMA/nanoparticles matrix after heat-polymerisation. Conversely, aggregation of the filler can block light transmission almost entirely, resulting in a significant change in colour or translucency [33]. Silanisation is another method for improving filler dispersion and enhancing translucency and mechanical properties [10]. In this study, 50% of the bioactive glass was silanised. This process can replace the interaction between the –OH groups of the glass fillers and the hydrolysable groups of the silane coupling agent, increasing the distance between the fillers and PMMA, allowing light to pass through more effectively [10].

Furthermore, the BAGs consist of particles below 1 µm in size and were subjected to ball milling for improved dispersion. However, achieving consistent changes in translucency for all specimens remained challenging. Despite employing a ball mill and partially silanised filler, obtaining uniform distribution throughout the specimen volume proved difficult, as depicted in Figure 3 and Figure 4. This observation underscores the crucial role of the refractive index in translucency. Changes in translucency may be linked to absorbed water, which could impede the passage of UV light and result in lower readings, indicating reduced translucency [34]. This phenomenon was evident in our investigation after immersing the specimens in distilled water, and it was confirmed for all PMMA specimens with the addition of bioactive glasses stored in distilled water. For example, the incorporation of 45S5 glass resulted in a change in translucency from 9.14 ± 0.93 to 8.91 ± 0.75, although these values were not statistically significant.

When the composition of the specimens was analysed using EDX, approximately 3% of calcium ions were observed. This indicates that the material releases these ions into the solution, leading to an increase in pH. Notably, the 45S5 glass exhibited a particularly high concentration of Ca^2+^ ions, which corresponded to the highest pH value in the study.

In their research, Rismanchian, et al. [34] present the cytotoxicity of bioactive glasses containing two types of nano and microsized glasses (35.42 mol% SiO_2_, 57.44 mol% CaO, and 7.15 mol% P_2_O_5_). At a concentration of ≥5 mg/mL in the first 48 h of applications, they observed cytotoxic properties towards human gingival fibroblasts. However, different conclusions were reached by Chen, et al. [35], who studied the influence of osteoblast cell cultures (MC3T3) on cell line 58S glass (60% SiO_2_, 36% CaO, 4% P_2_O_5_). In this case, the survival rate of cells in direct contact was 78% [35,36]. The bioactive material consisting of acrylic resin and containing 3% Novaron bioceramics, according to the authors of Chen, et al. [35], is not cytotoxic.

In these tests, high cell biocompatibility was achieved by incorporating all bioactive glasses into an acrylic block. PMMA-based resins do not exhibit cytotoxic properties if they are properly polymerised and have a low residual monomer content [1,37,38]. The addition of bioactive glass to such material results in partial hydrolysis of the glass due to water absorption. The appropriate concentration of ions is then delivered to the material’s surface, creating favourable conditions for cell proliferation. All specimens of materials containing bioactive glasses and PMMA showed no inhibition in cell cultures, both in MTT and Presto Blue assays. Furthermore, the Presto Blue assay, which is more sensitive than MTT, revealed increased cellular proliferation after exposure to 20% S53P4 and 20% Biomin F. However, it is important to note that the research conducted has certain limitations, as the tests were performed only for one type of acrylic resin. In the future, it is necessary to test other acrylic resins with different compositions and conduct additional biological tests, such as irritants, subcutaneous implants, and others.

## 5. Conclusions

1. The acrylic material fabricated with 20 wt.% bioactive glasses (S53P4, Biomin F, Biomin C, and 45S5) can be used to produce a removable denture to facilitate ion release.
2. The tested bioactive glasses contain calcium, aluminium, silicon, phosphorus, and sodium ions. Additionally, Biomin F contains fluoride ions.
3. These series of tests indicate that PMMA containing glass S53P4 can increase the pH of water solutions from 5.5 to 7.54 after 10 days.
4. The composite materials do not inhibit the growth of cell cultures from human fibroblasts.
5. Specimens containing pure PMMA have the highest transparency. However, the addition of bioactive substances reduces this property. When stored in water, the transparency is further reduced.
6. The roughness of the specimen slightly increases after the addition of bioactive glasses.

## Figures and Tables

**Table 1:** The composition of BAGs used in this study according to the information provided by the suppliers.

Bioactive Glasses	SiO_2_	P_2_O_5_	CaO	Na_2_O	CaF_2_	CaCl_2_
S53P4	53.8%	1.7%	21.8%	22.7%	0	0
Biomin F	36–40%	4–6%	28–30%	22–24%	1.5–3.0%	0
45S5	46.1%	2.6%	26.9%	24.4%	0	0
Biomin C	30.3–31.8%	5.0–5.3%	44.1–46.3%	0	0	16.7–20.6%

**Table 2:** Surface roughness (Ra μm) and translucency (TP) of the tested materials in dry condition and after 7 d of water storage.

Materials	Surface Roughness (Ra μm)		Translucency (TP)	
	Dry	Water (7 d)	Dry	Water (7 d)
PMMA	0.291 (0.03) ^ABa^	0.285 (0.04) ^Aa^	12.42 (1.59) ^Aa^	12.23 (1.37) ^Aa^
PMMA + Biomin F	0.268 (0.06) ^Aa^	0.273 (0.07) ^Aa^	8.15 (0.85) ^Ba^	7.87 (0.73) ^Ba^
PMMA + Biomin C	0.318 (0.04) ^BCa^	0.306 (0.05) ^Aa^	4.18 (0.39) ^Ca^	4.40 (0.50) ^Ca^
PMMA + S53P4	0.364 (0.04) ^CDa^	0.372 (0.06) ^Ba^	10.51 (1.33) ^Da^	10.19 (0.88) ^Da^
PMMA + 45S5	0.395 (0.05) ^Da^	0.402 (0.07) ^Ba^	9.14 (0.93) ^BDa^	8.91 (0.75) ^BDa^

In every column, distinct superscript capital letters denote significant differences between the materials and pure PMMA, which is used as a reference specimen (*p* < 0.05). In every row, identical superscript lowercase letters indicate no significant differences between dry/7 d within a tested material (*p* > 0.05).

**Table 3:** The composition of particles in individual glasses mixed in PMMA [wt.%].

	C	O	Cl	Ca	Zn	Al	Si	S	Fe	F	P	Na
PMMA	65.65	33.27	0.07	0.23	0.26	0.17	0.11	0.04	0.22			
20% Biomin F	59.85	33.84		3.08		0.26	1.43		0.22	0.24	0.43	0.65
20% Biomin C	61.72	35.00		2.09		0.16	0.36				0.48	0.19
20% 45S5	57.56	34.37	0.91	3.97		0.24	1.35		0.80		0.62	0.17
20% S53P4	58.22	35.02	0.06	3.44	0.22	0.22	1.42		0.52		0.42	0.46

## Data Availability

The data presented in this study are available in this article.

## References

[1] Sabri B., Satgunam M., Abreeza N.M., Abed A. (2021). A review on enhancements of PMMA Denture Base Material with Different Nano-Fillers. Cogent Eng..

[2] Lourinho C., Salgado H., Correia A., Fonseca P. (2022). Mechanical Properties of Polymethyl Methacrylate as Denture Base Material: Heat-Polymerized vs. 3D-Printed-Systematic Review and Meta-Analysis of In Vitro Studies. Biomedicines.

[3] Giti R., Zomorodian K., Firouzmandi M., Zareshahrabadi Z., Rahmannasab S. (2021). Antimicrobial Activity of Thermocycled Polymethyl Methacrylate Resin Reinforced with Titanium Dioxide and Copper Oxide Nanoparticles. Int. J. Dent..

[4] Tsutsumi C., Takakuda K., Wakabayashi N. (2016). Reduction of Candida Biofilm Adhesion by Incorporation of Prereacted Glass Ionomer Filler in Denture Base Resin. J. Dent..

[5] Gligorijević N., Mihajlov-Krstev T., Kostić M., Nikolić L., Stanković N., Nikolić V., Dinić A., Igić M., Bernstein N. (2022). Antimicrobial Properties of Silver-Modified Denture Base Resins. Nanomaterials.

[6] Sun J., Wang L., Wang J., Li Y., Zhou X., Guo X., Zhang T., Guo H. (2021). Characterization and evaluation of a novel silver nanoparticles-loaded polymethyl methacrylate denture base: In vitro and in vivo animal study. Dent. Mater. J..

[7] Alhotan A., Yates J., Zidan S., Haider J., Silikas N. (2021). Assessing fracture toughness and impact strength of PMMA reinforced with nano-particles and fibre as advanced denture base materials. Materials.

[8] Totu E.E., Nechifor A.C., Nechifor G., Aboul-Enein H.Y., Cristache C.M. (2017). Poly (methyl methacrylate) with TiO2 nanoparticles inclusion for stereolithographic complete denture manufacturing—The future in dental care for elderly edentulous patients?. J. Dent..

[9] Bacali C., Baldea I., Moldovan M., Carpa R., Olteanu D.E., Filip G.A., Nastase V., Lascu L., Badea M., Constantiniuc M. (2020). Flexural strength, biocompatibility, and antimicrobial activity of a polymethyl methacrylate denture resin enhanced with graphene and silver nanoparticles. Clin. Oral Investig..

[10] Kamonkhantikul K., Arksornnukit M., Takahashi H. (2017). Antifungal, Optical, and Mechanical Properties of Polymethylmethacrylate Material Incorporated with Silanized Zinc Oxide Nanoparticles. Int. J. Nanomed..

[11] Gad M.M., Alshehri S.Z., Alhamid S.A., Albarrak A., Khan S.Q., Alshahrani F.A., Alqarawi F.K. (2022). Water Sorption, Solubility, and Translucency of 3D-Printed Denture Base Resins. Dent. J..

[12] Hamid S.K., Alghamdi L.A., Alshahrani F.A., Khan S.Q., Matin A., Gad M.M. (2021). In Vitro Assessment of Artificial Aging on the Antifungal Activity of PMMA Denture Base Material Modified with ZrO2 Nanoparticles. Int. J. Dent..

[13] Alzayyat S.T., Almutiri G.A., Aljandan J.K., Algarzai R.M., Khan S.Q., Akhtar S., Matin A., Gad M.M. (2021). Antifungal Efficacy and Physical Properties of Poly(Methylmethacrylate) Denture Base Material Reinforced with SiO2 Nanoparticles. J. Prosthodont..

[14] Bajunaid S.O., Baras B.H., Weir M.D., Xu H.H. (2022). Denture Acrylic Resin Material with Antibacterial and Protein-Repelling Properties for the Prevention of Denture Stomatitis. Polymers.

[15] An J., Ding N., Zhang Z. (2022). Mechanical and antibacterial properties of polymethyl methacrylate modified with zinc dimethacrylate. J. Prosthet. Dent..

[16] Da Silva Barboza A., Fang L.K., Ribeiro J.S., Cuevas-Suárez C.E., Moraes R.R., Lund R.G. (2021). Physicomechanical, Optical, and Antifungal Properties of Polymethyl Methacrylate Modified with Metal Methacrylate Monomers. J. Prosthet..

[17] An S., Evans J.L., Hamlet S., Love R.M. (2021). Incorporation of antimicrobial agents in denture base resin: A systematic review. J. Prosthet. Dent..

[18] Raszewski Z., Chojnacka K., Mikulewicz M., Alhotan A. (2023). Bioactive Glass-Enhanced Resins: A New Denture Base Material. Materials.

[19] Asgari N., Baaske M., Orrit M. (2023). Burst-by-Burst Measurement of Rotational Diffusion at Nanosecond Resolution Reveals Hot-Brownian Motion and Single-Chain Binding. ACS Nano.

[20] Gusmão G.M., De Queiroz T.V., Pompeu G.F., Menezes Filho P.F., da Silva C.H. (2013). The influence of storage time and pH variation on water sorption by different composite resins. Indian J. Dent. Res..

[21] Moslehifard E., Ghaffari T., Abolghasemi H., Maleki Dizaj S. (2022). Comparison of Conventional Pressure-packed and Injection Molding Processing Methods for an Acrylic Resin Denture based on Microhardness, Surface Roughness, and Water Sorption. Int. J. Dent..

[22] Alshamrani A., Alhotan A., Owais A., Ellakwa A. (2023). The Clinical Potential of 3D-Printed Crowns Reinforced with Zirconia and Glass Silica Microfillers. J. Funct. Biomater..

[23] Nowakowska D., Saczko J., Kulbacka J., Choromanska A., Raszewski Z. (2012). Cytotoxic potential of vasoconstrictor experimental gingival retraction agents: In vitro study on primary human gingival fibroblasts. Folia Biol..

[24] Saczko J., Dominiak M., Kulbacka J., Chwiłkowska A., Krawczykowska H. (2008). A simple and established method of tissue culture of human gingival fibroblasts for gingival augmentation. Folia Histochem. Cytobiol..

[25] Elmergawy F.H., Nassif M.S., El-Borady O.M., Mabrouk M., El-Korashy D.I. (2021). Physical and mechanical evaluation of dental resin composite after modification with two different types of Montmorillonite nanoclay. J. Dent..

[26] Gad M.M., Abu-Rashid K., Alkhaldi A., Alshehri O., Khan S.Q. (2022). Evaluation of the effectiveness of bioactive glass fillers against Candida albicans adhesion to PMMA denture base materials: An in vitro study. Saudi Dent. J..

[27] Berger J.C., Driscoll C.F., Romberg E., Luo Q., Thompson G. (2006). Surface roughness of denture base acrylic resins after processing and after polishing. J. Prosthodont..

[28] Costa R.T.F., Pellizzer E.P., Vasconcelos B., Gomes J.M.L., Lemos C.A.A., de Moraes S.L.D. (2021). Surface roughness of acrylic resins used for denture base after chemical disinfection: A systematic review and meta-analysis. Gerodontology.

[29] Al-Rifaiy M.Q. (2010). The effect of mechanical and chemical polishing techniques on the surface roughness of denture base acrylic resins. Saudi Dent. J..

[30] Xu X., Burgess J.O. (2003). Compressive strength, fluoride release and recharge of fluoride-releasing materials. Biomaterials.

[31] Sagadevan K.S.S., Ravichandran R., Harsha Kumar K., Nair V.V., Kavitha J., Deepthi V. (2021). Effect of zirconium oxide and cellulose nanoparticles addition on the flexural strength, impact strength and translucency of heat polymerized acrylic resin: An in vitro study. Int. J. Dent. Mater..

[32] Hamid S.K., Al Dubayan A.H., Alghamdi L.A. (2021). Mechanical, Surface, and Optical Properties of PMMA Denture Base Material Modified with Azadirachta indica as an Antifungal Agent. J. Contemp. Dent. Pract..

[33] Gad M.M., Abualsaud R., Alqarawi F.K., Emam A.N.M. (2021). Translucency of nanoparticle-reinforced PMMA denture base material: An invitro comparative study. Dent. Mater. J..

[34] Rismanchian M., Khodaeian N., Bahramian L., Fathi M., Sadeghi-Aliabadi H. (2013). In-vitro Comparison of Cytotoxicity of Two Bioactive Glasses in Micropowder and Nanopowder forms. Iran. J. Pharm. Res..

[35] Chen R., Han Z., Huang Z., Karki J., Wang C., Zhu B., Zhang X. (2017). Antibacterial activity, cytotoxicity and mechanical behavior of nano-enhanced denture base resin with different kinds of inorganic antibacterial agents. Dent. Mater. J..

[36] Safwat E.M., Alkabani Y.M., Zaki D.Y. (2023). Preparation and Characterization of Dental Pit and Fissure Sealant Based on Calcium Sodium Silicate Bioactive Glasses. Silicon.

[37] Chen J., Zeng L., Chen X., Liao T., Zheng J. (2017). Preparation and characterization of bioactive glass tablets and evaluation of bioactivity and cytotoxicity in vitro. Bioact. Mater..

[38] Salehi S., Gwinner F., Mitchell J.C., Pfeifer C., Ferracane J.L. (2015). Cytotoxicity of resin composites containing bioactive glass fillers. Dent. Mater. J..

